# Joint Sparsity Constraint Interferometric ISAR Imaging for 3-D Geometry of Near-Field Targets with Sub-Apertures

**DOI:** 10.3390/s18113750

**Published:** 2018-11-02

**Authors:** Yang Fang, Baoping Wang, Chao Sun, Shuzhen Wang, Jiansheng Hu, Zuxun Song

**Affiliations:** 1 School of Electronics and Information, Northwestern Polytechnical University, Xi’an 710072, China; sunchao2013@mail.nwpu.edu.cn (C.S.); zxsong@nwpu.edu.cn (Z.S.); 2 National Key Laboratory of Science and Technology on UAV, Northwestern Polytechnical University, Xi’an 710065, China; 3 School of Computer Science and Technology, Xidian University, Xi’an 710071, China; 4 Department of Information Engineering, PAP of Engineering University, Xi’an 710068, China; hujiansheng121@163.com

**Keywords:** joint sparse reconstruction, interferometric inverse synthetic aperture radar, compressed sensing, near-field 3-D imaging, wide angle

## Abstract

This paper proposes a new interferometric near-field 3-D imaging approach based on multi-channel joint sparse reconstruction to solve the problems of conventional methods, i.e., the irrespective correlation of different channels in single-channel independent imaging which may lead to deviated positions of scattering points, and the low accuracy of imaging azimuth angle for real anisotropic targets. Firstly, two full-apertures are divided into several sub-apertures by the same standard; secondly, the joint sparse metric function is constructed based on scattering characteristics of the target in multi-channel status, and the improved Orthogonal Matching Pursuit (OMP) method is used for imaging solving, so as to obtain high-precision 3-D image of each sub-aperture; thirdly, comprehensive sub-aperture processing is performed using all sub-aperture 3-D images to obtain the final 3-D images; finally, validity of the proposed approach is verified by using simulation electromagnetic data and data measured in the anechoic chamber. Experimental results show that, compared with traditional interferometric ISAR imaging approaches, the algorithm proposed in this paper is able to provide a higher accuracy in scattering center reconstruction, and can effectively maintain relative phase information of channels.

## 1. Introduction

Near-field 3-D imaging is a microwave imaging technique that is developed on the basis of two-dimensional (2-D) synthetic aperture imaging. As it has higher spatial resolution capability, and has easy availability for engineering realization, near-field 3-D imaging is widely applied in Radar Cross Section (RCS) [1], non-destructive testing and evaluation (NDTE) [2,3], security check [4], concealed weapon detection [5,6,7], through-wall and inner wall imaging [8,9], breast cancer detection [10,11], etc. So far, a variety of techniques have been applied in near-field 3-D imaging to improve its performance, such as imaging based on range migration algorithm (RMA) [12,13,14] and polar format algorithm (PFA) [14], tomography imaging method [15], microwave holography method [16], confocal radar-based imaging [17], and NUFFT-based imaging [18,19].

Imaging approaches as mentioned above are all based on the traditional Nyquist sampling principle and matched filtering. In general, high range resolution is obtained by transmitting wideband signals, and high azimuth resolution is obtained in longer synthetic aperture time. However, with higher requirements for imaging resolution, traditional imaging approaches are encountering problems such as the sampling rate is too high, the data volume is too large, and the fast processing is difficult to carry out. According to the Compressed Sensing (CS) [20,21], a sparse signal or a sparse signal in a particular transform domain is sampled in a way that is lower than or far below than requirements of the Nyquist sampling theorem. The high-dimensional target signal can be accurately reconstructed by applying low-dimensional observation data by solving a minimum L-norm constrained optimization problem. At present, many scholars are combining CS with high-resolution near-field imaging and have achieved a number of research results, which fully demonstrate great potential of CS in reducing data sampling rate and improving imaging resolution [3,8,22,23].

At present, research on near-field 3-D imaging mainly focuses on planar scanning 3-D imaging. Although high-resolution target 3-D images can be obtained by applying the planar scanning 3-D imaging, the size of sampling data is huge, and conducting the measurement is time-consuming and the imaging efficiency is low. For the interferometric inverse synthetic aperture radar (InISAR) [24,25] method, 3-D views of the target can be obtained by applying the multi-antenna phase interference method, and data acquisition and signal processing are relatively simple, which make it easy for the system to perform functions. Thus, InISAR can be widely used in near-field 3-D imaging. InISAR 3-D imaging based on CS technology has the following advantages: (1) high-resolution ISAR images can be obtained only by short-time observation data. At the same time, rotation of the target can be approximately considered to be uniform in short-phase processing interval and thus the occurrence probability of range cell migration is reduced; (2) imaging results are not affected by sidelobe, and image resolution can be improved by increasing imaging grids, so that it is helpful to suppress angular glint phenomenon in InISAR imaging and (3) through the CS technology, the ISAR images can be further reconstructed by adopting sparse sampling data, thus reducing the pressure of data acquisition.

In interferometric imaging, compressed sampling and sparse reconstruction can be separately performed for each channel, so as to reduce the system sampling rate and improve the quality of radar imaging. For example, 3-D InISAR imaging method based on sparse constraint model is proposed using the sparsity of ISAR images in reference [24]. However, traditional InISAR imaging methods have the following problems: (1) because the observation objectives are consistent, multi-channel echoes in the InISAR system have a strong correlation, i.e., images of each channel have the same target support set. However, in single-channel independent processing, such prior information is not considered, and consistent location and number of scattering points among channel images cannot be ensured, which means it cannot ensure that all scattering points on the target are located in positions with the same pixel in two interferometric images, thus reducing the estimation accuracy of interferometric phase information and (2) in InISAR imaging, scattering characteristics of the target vary with the observation angle, and the imaging azimuth accuracy is limited by scattering anisotropy of the target.

Motived by the above problems in traditional InISAR imaging methods, this paper proposes the interferometric near-field 3-D imaging based on multi-channel joint sparse reconstruction. Firstly, a more universal multi-channel interferometric near-field echo signal model is set up; secondly, the two observed full apertures are divided into several sub-apertures according to the same criteria. By analyzing sparse characteristics of the target echo in each channel, a joint sparse constrained optimization model is set up and the problem of multi-channel high-resolution imaging is transformed into an optimization problem based on multi-channel joint sparse reconstruction. The improved orthogonal matching pursuit (OMP) is applied for high-resolution imaging solving to obtain 3-D images of each sub-aperture target; thirdly, the 3-D images of each sub-aperture are synthesized to obtain final 3-D imaging results of the target under full aperture; finally, the effectiveness of the proposed approach is verified by processing point target simulation data and Backhoe electromagnetic simulation data, and the InISAR system is set up in the microwave anechoic chamber to verify the practical applications of the proposed approach by processing the measured data obtained. Compared with traditional InISAR imaging methods, the proposed method in this paper has the following advantages: (1) reconstruction accuracy of strong scattering centers in ISAR images is improved owing to utilization of correlation among cross channels, and relative phase information of cross channels is kept effectively, so as to obtain interferometric phase information with higher accuracy; (2) owing to sub-aperture synthesis method applied, the target with scattering anisotropy in all directions can be accurately described, and the problem that accuracy of imaging azimuth angle is limited can be overcome; (3) because of information complementation and redundancy among multi-channel signals applied, given relatively large compression sampling ratio, generation of false scattering points can be effectively suppressed, thereby improving the imaging quality.

## 2. Signal Model of Inisar Near-Field Imaging

**Figure 1:**
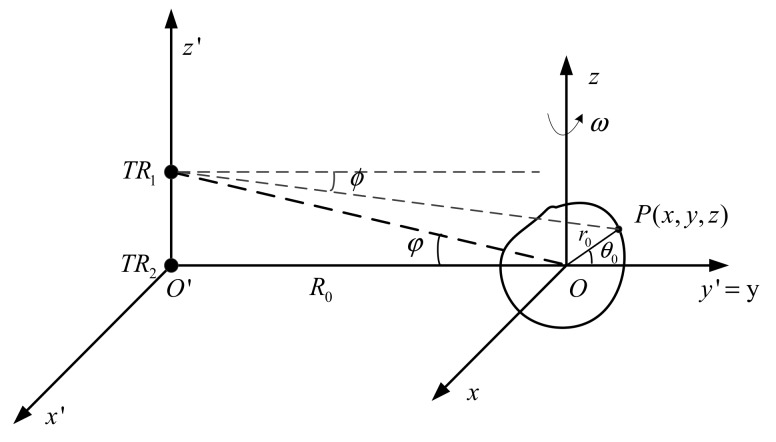
Geometric sketch of dual-antenna InISAR imaging.

The InISAR system includes multiple antennas. This paper proposes a dual-antenna ISAR imaging system. Figure 1 shows geometric relationship between antennas and the target, where antenna *TR*_2_ is located at origin *O’*. Antennae *TR*_1_ and *TR*_2_ form the vertical baseline along axis *Z’*. Define two coordinate systems, where *T’*(*x’,y’,z’*) is the radar coordinate system, axis *y’* is the line of sight of radar, *x*’ and *y*’ represent the horizontal and vertical directions, respectively. *T*(*x,y,z*) is the target coordinate system, in which axis *y* is coincident with axis *y’*, and axes *x* and *y* represent the azimuth direction and range direction of ISAR, respectively, and distance between origins of the two coordinate systems is *R*_0_ (*R*_0_ < 4*D*^2^/*λ*, *D* is the maximum size of the target and *λ* is the wavelength of the incident wave). The target is moving at a constant speed in plane (*x,y*) at an angular velocity *ω*, and plane (*x’,y’*) is parallel to plane (*x,y*). Assuming that coordinate of any point *P* on the target is (*x,y,z*), and the coordinate in the cylindrical coordinate system is (*r*_0_,*θ*_0_,*z*). Then, at the moment *t*, the distance from the antenna *I* (*i*∈ *TR*_1_, *TR*_2_}) to the point *P* is:

(1) $$\begin{array}{l} R_{i}(t)=\sqrt{{\left(R_{0}+y\right)}^{2}+x^{2}+{\left(R_{0}\tan\alpha_{i}-z\right)}^{2}}\\ =\sqrt{{\left(R_{0}+r_{0}\cos(\theta_{0}+\omega t)\right)}^{2}+{\left(r_{0}\sin(\theta_{0}+\omega t)\right)}^{2}+{\left(R_{0}\tan\alpha_{i}-z\right)}^{2}} \end{array}$$

where, *α_i_* refers to the pitch angle from the antenna *i* to the origin of target coordinate system. Assuming that the antenna transmits a step frequency wideband signal [26]:

(2) $$s_{it}(t)=\sum_{m=0}^{M-1}\mathrm{rect}\left(\frac{t-t_{mp}}{T}\right)\exp[j2\pi f_{m}t]$$

where:

(3) $$\mathrm{rect}\left(\frac{t-t_{mp}}{T}\right)=\left\{ \begin{array}{l} 1,\;0<\frac{t-t_{mp}}{T}<1;\\ 0,\;\frac{t-t_{mp}}{T}<0\;and\;\frac{t-t_{mp}}{T}>1; \end{array}\right.$$

$f_{m}=f_{0}+\Delta f$ is the frequency of the pulse centered at time $t_{mp}$ and for pulses spaced equally in frequency and time; $t_{mp}=(m+pM)T$; Δf is the frequency difference for each step in the pulse burst; *T* is the time interval between pulses (pulse repetition period of pulses in the burst); *M* the number of pulses in each burst; $p=\bar{0,N-1}$-the index of emitted burst. The echo of the target received by antenna i after the coherent demodulation is (to understand easily, rectangular coordinates are used):

(4) $$s_{ir}(t)=\iint_{D}g_{i}(x,y)\sum_{m=0}^{M-1}\mathrm{rect}\left(\frac{t-t_{mp}}{T}\right)\exp[j2\pi f_{m}\frac{R_{i}(t,x,y)}{c}]dxdy,$$

where, D refers to the imaging scene area, (x,y) refers to the position coordinate of the target, and $g_{i}(x,y)$ indicates the backscatter coefficient of the target at position (x,y) received by antenna i. Since the imaging process is a linear system, and in the high frequency region, the total scattering of the target can be seen as a linear superposition of multiple strong scattering points, i.e., $s_{ir}(t)$ represents superposition of echo signals of all scattering points at (x,y) in the entire imaging region $g_{i}(x,y)$. The signal is a complex signal, amplitude $g_{i}(x,y)$ represents scattering intensity of scattering point (x,y), and phase $\exp(-j4\pi f_{m}R_{i}(t,x,y)/c)$ contains position information (x,y) of the scattering point. $\iint_{D}[\cdot ]dxdy$ represents the summation of all the scattered echoes in the imaging scene. With signal processing technology, the target image can be obtained by separating position (x,y) and amplitude $g_{i}(x,y)$ from complex signal $s_{ir}(t)$.

The imaging scene is discretized. In order to represent the radar signal as a matrix, the corresponding 2-D backscatter coefficient matrix is concatenated into a one-dimensional column vector by row or line:

(5) $$g_{i}={\left[g_{i}(1,1),\cdots ,g_{i}(P,1),g_{i}(1,2),\cdots ,g_{i}(P,2),\cdots ,g_{i}(1,Q),\cdots ,g_{i}(P,Q)\right]}^{T},$$

where, $g_{i}$ refers to the vector of PQ×1, and P is the discrete grid number of axis x. Q is the discrete grid number of axis y.

According to the Formula (4), the discrete echo data can be indicated as follows:

(6) $$s_{ir}\left(t\right)=\sum_{l=1}^{PQ}\sum_{m=0}^{M-1}g_{i}(l)\exp\left[-j\frac{4\pi }{\lambda_{m}}R_{i}\left(t,l\right)\right],$$

where, $\lambda_{m}$ refers to wave length corresponding to the frequency $f_{m}$. $R_{i}\left(t,l\right)$ refers to the distance between the ith antenna and the lth target at time *t*.

The range (or frequency) and azimuth angle are also discrete in actual situations. Assuming that the range sampling point is N, the azimuth sampling point is M. The frequency of nth (frequency) pulse in the burst is defined by $f_{n}=f_{0}+(n-1)\Delta f,n=1,2,\cdots ,N$, where $f_{0}$ is the initial frequency, Δf is the step interval, and the azimuth is discretized as $\theta_{m}=(m-1)\Delta \theta ,m=1,2,\cdots ,M$, (Δθ is the angular interval). Formula (6) can be discretized as:

(7) $$s_{ir}\left(f_{n},\theta_{m}\right)=\sum_{l=1}^{PQ}g_{i}(l)\exp\left[-j\frac{4\pi f_{n}}{c}R_{i}\left(\theta_{m},l\right)\right]$$

Equation (7) is represented as a matrix considering the effect of noise in actual situations:

(8) $$s_{ir}=A_{i}g_{i}+e_{i},$$

where $s_{i}$ is a vector with the size of MN×1, which is formed through signal sampling; $A_{i}$ is a dictionary matrix with the size of MN×PQ, which is formed through mapping relationship between the target and the signal; $g_{i}$ is a vector with the size of PQ×1, which is composed of scene backscatter coefficients; $e_{i}$ is the additive complex noise in the channel. Specified composition of each vector and matrix in formula (8) is as follows:

(9) $$\begin{array}{ll} s_{ir} & =[s_{ir}\left(f_{1},\theta_{1}\right),\cdots ,s_{ir}\left(f_{1},\theta_{M}\right),s_{ir}\left(f_{2},\theta_{1}\right),\cdots ,\\ & s_{ir}\left(f_{2},\theta_{M}\right),\cdots s_{ir}\left(f_{N},\theta_{1}\right),\cdots ,s_{ir}\left(f_{N},\theta_{M}\right)] \end{array}$$

Make:

(10) $$a(f_{n},\theta_{m},l)=\exp\left[-j\frac{4\pi f_{n}}{c}R_{i}\left(\theta_{m},l\right)\right]$$

Following vectors are defined as:

(11) $$a(f_{n},\theta_{m})={\left[a(f_{n},\theta_{m},1),a(f_{n},\theta_{m},2),\cdots ,a(f_{n},\theta_{m},PQ)\right]}^{T}.$$

Then the dictionary matrix can be obtained as:

(12) $$A_{i}=\left[\alpha (f_{1},\theta_{1}),\cdots ,\alpha (f_{1},\theta_{M}),\alpha (f_{2},\theta_{1}),\cdots ,\alpha (f_{2},\theta_{M}),\cdots ,\alpha (f_{N},\theta_{1}),\cdots ,\alpha (f_{N},\theta_{M})\right]$$

Therefore, the projection relationship between the scene and signal in i channel is obtained. The radar signal model in the interferometric channel is represented as follows:

(13) $$s=\left[\begin{array}{l} s_{1r}\\ s_{2r} \end{array}\right]=Ag+e=\left[\begin{array}{l} A_{1}0\\ 0A_{2} \end{array}\right]\left[\begin{array}{l} g_{1}\\ g_{2} \end{array}\right]+\left[\begin{array}{l} e_{1}\\ e_{2} \end{array}\right],$$

where, s corresponds with the echo signal in the interferometric channel, A corresponds with the dictionary matrix in the interferometric channel, g corresponds with the backscatter coefficient in each interferometric channel, and e corresponds with the additive noise in each interferometric channel. It should be noted that the dictionary matrix corresponding with each interferometric channel may be the same. However, in order to keep their generality, dictionary matrixes of the two interferometric channels should be represented separately.

In order to reduce the sampling data of each interferometric channel effectively, the $M^{\prime }(M^{\prime }\leq M)$ angular position is randomly selected to transmit signal in the azimuth direction, and then $N^{\prime }(N^{\prime }\leq N)$ frequency point is randomly selected in the distance direction. After compressed sampling, the interferometric echo signal model can be represented as follows:

(14) $$s^{\prime }=\Phi s=\left[\begin{array}{l} \Phi_{1}0\\ 0\Phi_{2} \end{array}\right]s=\Phi Ag+\Phi e=A^{\prime }g+e^{\prime },$$

where, $s^{\prime }$ refers to CS echo signal with the size of $2N^{\prime }M^{\prime }\times 1$, $\Phi_{i}=\Phi_{i}^{a}⊗\Phi_{i}^{r}$ refers to the measurement matrix corresponding with each channel, ⊗ refers to the Kronecker product, $A^{\prime }$ refers to the sensing matrix with the size of $2N^{\prime }M^{\prime }\times PQ$, g refers to the scene backscatter coefficient with the size of 2PQ×1, and $e^{\prime }$ refers to noise with the size of 2PQ×1.

Since $M^{\prime }\leq M$, $N^{\prime }\leq N$, recovery of the signal s from the measurements $s^{\prime }$ is ill-posed in general. However, according to the CS theory, when the matrix $\Phi A=A^{\prime }$ has the Restricted Isometry Property (RIP) [27], it is indeed possible to recover the K largest $g_{i}$’s from a similarly sized set of $M^{\prime }N^{\prime }=O(K\log(MN/K))$ measurements $s^{\prime }$. The RIP is closely related to an incoherency property between Φ and A, where the rows of Φ do not provide a sparse representation of the columns of A, and vice versa.

In order to obtain the target images of each channel, the problem of interferometric near-field imaging can be converted into an optimization and reconstruction problem of two independent channels according to CS theory and sparse space distribution characteristics of the target scene:

(15) $$\left\{ \begin{array}{l} \min{\left\|g_{1}\right\|}_{0\;}s.t.\;{\left\|s\prime_{1}-A\prime_{1}g_{1}\right\|}_{F}\leq \xi_{1}\\ \min{\left\|g_{2}\right\|}_{0\;}s.t.\;{\left\|s\prime_{2}-A\prime_{2}g_{2}\right\|}_{F}\leq \xi_{2} \end{array}\right.$$

where, ${\left\|•\right\|}_{0}$ refers to the zero norm of vector, namely the number of non-zero elements in the vector, ${\left\|•\right\|}_{F}$ refers to the Frobenius norm of matrix, and $\xi_{i}$ is a positive number which depends on the noise level. In Formula (15), it is quite important to select an appropriate noise level for the final optimization result: too high noise level will lead to the loss of some weak scattering points, while too low noise level will make it difficult to suppress strong noises. In Formula (15), optimization solving can be performed by applying the OMP method which not only guarantees reconstruction accuracy, but also has high computational efficiency.

## 3. Joint Sparsity Constraint Near-Field 3-D Imaging of Inisar Based on CS

### 3.1. Algorithm Flow Description

**Figure 2:**
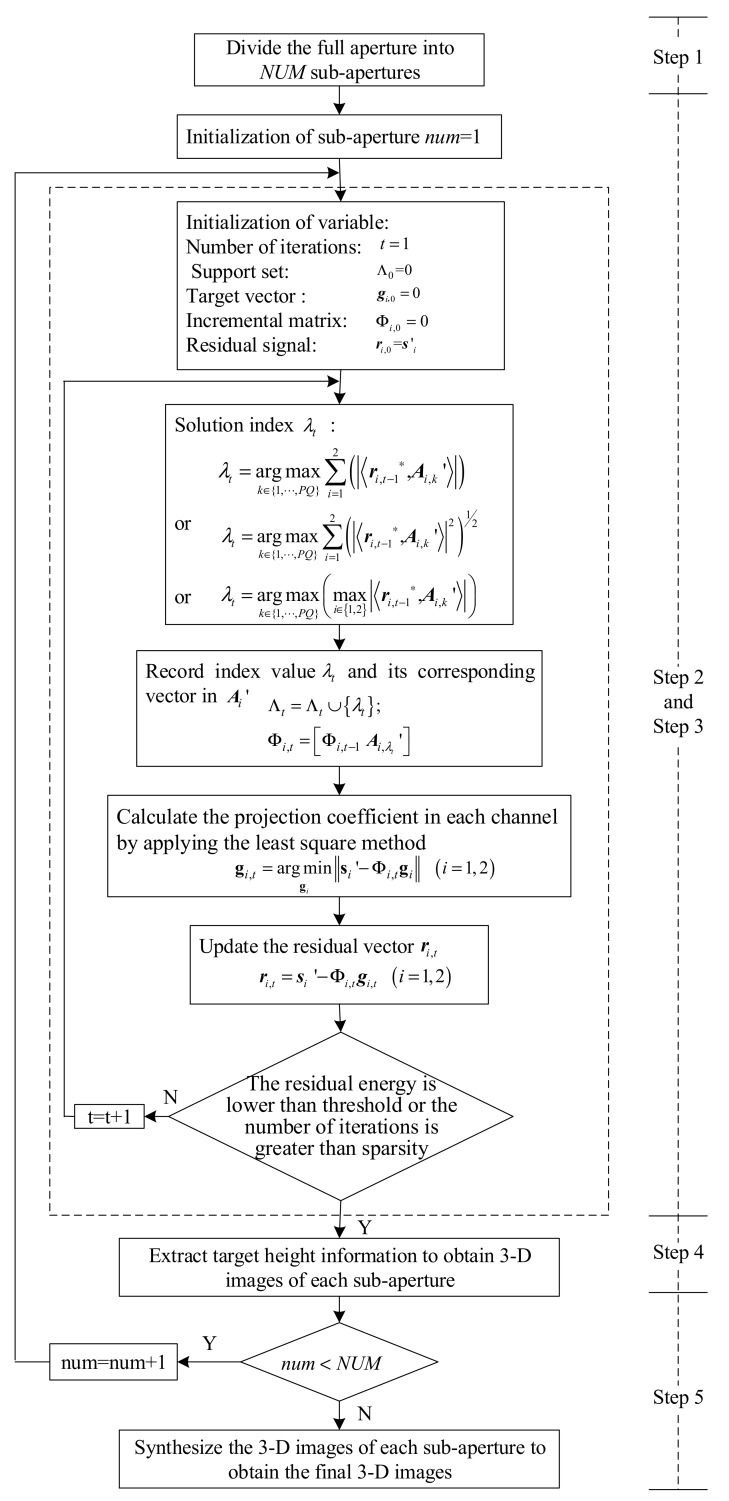
Imaging flow of proposed approach.

We first give the flow of the proposed approach in this paper, and describe the solution precisely in the following subsections. The process of the proposed approach is presented as follows:

Step 1: Full apertures of the two channels are divided into several sub-apertures by the same criteria, and each sub-aperture has a very small azimuth angle range.
Step 2: Global sparsity constraint and improved OMP algorithm are applied to obtain the 2-D complex images $I_{1}$ and $I_{2}$ of each sub-aperture in the two channels.
Step 3: Two images of each sub-aperture are performed with interferometric processing to obtain projection coordinates of the scattering points along the baseline.
Step 4: 3-D images of each sub-aperture target are constructed by synthesizing the 2-D ISAR images of the interferometric processing results.
Step 5: 3-D images of all sub-apertures are processed synthetically to obtain the final 3-D images.
Step 6: The imaging flow is shown in Figure 2.

### 3.2. Joint Sparse Constrained Optimization Model

As mentioned in Section 2, a small amount of compressed sampled data is used to achieve high-resolution reconstruction of the scene in the single-channel independent CS approach. However, it cannot guarantee consistency of positions and numbers of all scattering points in each channel, and integrity of the cross-information of channels is destroyed, which are not favorable for target scattering information extraction. In addition, complementarity and redundancy among multi-channel data does not fully exploit in the single-channel independent CS approach, so it cannot further improve the SNR gain and reduce data volume of the system.

Two ISAR images used in the InISAR imaging are usually highly correlated, so they are more obvious in joint sparsity. Based on such prior information, the following joint sparse metric functions with global sparsity can be obtained as:

(16) ‖g‖p,0=‖(|g1|p+|g2|p)1p‖0,p≥1,

where, ${\left\|•\right\|}_{0}$ is zero norm, which represents the number of non-zero elements in the vector. Different p values correspond with different mixed norm forms. This paper defines the following three global sparsity constraints:

(1) when p=1, ${\left\|g\right\|}_{1,0}={\left\|\left(\left|g_{1}\right|+\left|g_{2}\right|\right)\right\|}_{0}$, is termed as mixed sum norm, i.e., sparsity of amplitude sum of the two ISAR images is taken as the global sparsity constraint;
(2) when p=2, ‖g‖2,0=‖(|g1|2+|g2|2)12‖0 is termed as mixed Euclidean norm;
(3) when p=∞, ${\left\|g\right\|}_{\infty ,0}={\left\|\max\left(\left|g_{1}\right|,\left|g_{2}\right|\right)\right\|}_{0}$ is termed as mixed infinite norm, i.e., the sparsity of one of the two ISAR images (with larger amplitude) is taken as the overall sparsity constraint.

The mixed sum norm and mixed Euclidean norm are both measured by taking the number of non-zero elements of amplitude sum of images in all channels as the global sparsity. Since the scattering points are aligned at different angles, the additivity is reasonable. The mixed infinite norm takes the number of non-zero elements of the pixel point with the largest amplitude in each image as the joint sparsity, and can also ensure that scattering points in the reconstructed sub-aperture images are aligned.

### 3.3. Optimal Solution Algorithm Constrained by Joint Sparse

According to the constructed global sparsity constraint function, the problem of InISAR imaging solution is transformed into the problem of multi-channel joint sparse optimal reconstruction problem:

(17) $$\underset{g_{1},g_{2}}{\min}{\left\|g\right\|}_{p,0}s.t.\left\{ \begin{array}{l} {\left\|s\prime_{1}-A\prime_{1}g_{1}\right\|}_{2}^{2}\leq \xi \\ {\left\|s\prime_{2}-A\prime_{2}g_{2}\right\|}_{2}^{2}\leq \xi \end{array}\right.,$$

where, ξ is determined by the minimum noise level of each channel to ensure that each channel can generate the target image. In Formula (17), data cannot be processed directly by applying the traditional CS optimal reconstruction algorithm. In [28], an improved convex optimization approach was proposed to solve the joint sparse imaging problem of interferometric channels. As sparse reconstruction is only performed for azimuth, the computational complexity is not high. While it is applied to the 2-D sparse reconstruction concerning range and azimuth as studied in this paper, the computational complexity becomes intensive, especially in high-resolution imaging. The target coefficient vector to be reconstructed usually has a large size, and thus the memory shortage of the computational platform will be inevitably encountered for practical applications. OMP algorithm is a commonly used greedy algorithm, which has high computational efficiency and can guarantee excellent reconstruction results. Therefore, this paper proposes an effective and improved OMP algorithm to solve the multi-channel joint sparse reconstruction problem based on OMP algorithm. Regarding the three mixed norm solutions proposed in this paper, step (2) is different in the way of index finding:

(1) Initialization: number of iterations t=1, and support set $\Lambda_{0}=0$. For the ith channel, its initialized target vector $g_{i,0}=0$, and incremental matrix $\Phi_{i,0}=0$, which is composed of column vectors in the support set. Make $r_{i,t}$ the residual signal after t iterations, and initialize $r_{i,0}=s\prime_{i}$.
(2) Obtain index $\lambda_{t}$ by solving the following formulas:
  (18) $$\mathrm{Mixed}\;\mathrm{sum}\;\mathrm{norm}\text{:}\;\lambda_{t}=\underset{k\in \{1,\cdots ,PQ\}}{\arg\;\max}\sum_{i=1}^{2}\left(\left|\langle r_{i,t-1}^{}{}^{*},A_{i,k}^{}\prime \rangle \right|\right),$$
  (19) Mixed Euclidean norm: λt=arg maxk∈{1,⋯,PQ}∑i=12(|〈ri,t−1∗,Ai,k′〉|2)12,
  (20) $$\mathrm{Mixed}\;\mathrm{infinite}\;\mathrm{norm}\text{:}\;\lambda_{t}=\underset{k\in \{1,\cdots ,PQ\}}{\arg\;\max}\left(\underset{i\in \left\{1,2\right\}}{\max}\left|\langle r_{i,t-1}^{}{}^{*},A_{i,k}^{}\prime \rangle \right|\right),$$
  where, $A_{i,k}^{}\prime$ is the k column vector in perception matrix.
(3) Record the obtained index $\lambda_{t}$ to the support set and its corresponding vector in $A_{i}\prime$ to the incremental matrix:
  (21) $$\begin{array}{l} \Lambda_{t}=\Lambda_{t}\cup \left\{\lambda_{t}\right\};\\ \Phi_{i,t}=\left[\Phi_{i,t-1}\;A_{i,\lambda_{t}}^{}\prime \right] \end{array}$$
(4) Adopt the least square method to calculate the projection coefficient of each channel:
  (22) $$g_{i,t}=\underset{g_{i}}{\arg\;\min}\left\|s_{i}\prime -\Phi_{i,t}g_{i}\right\|\left(i=1,2\right)$$
(5) Update residual signal $r_{i,t}^{}$:
  (23) $$r_{i,t}^{}=s_{i}^{}\prime -\Phi_{i,t}g_{i,t}\left(i=1,2\right)$$
(6) For the number of iteration t=t+1, repeat step (2) to (4) until the energy of the residual signal is lower than the preset threshold *Thres* or the number of iterations reaches the preset sparsity *K*.

Compared with the standard OMP algorithm, the proposed algorithm is mainly improved in step (2). In the standard OMP algorithm, the support set for different channels may be different, because the index $\lambda_{t,i}$ is determined only for the single-channel signal itself:

(24) $$\lambda_{t,l}=\underset{k\in \{1,\cdots ,PQ\}}{\arg\;\max}\langle r_{i,t-1}^{}{}^{*},A_{i,k}^{}\rangle \left(i=1,2\right).$$

Such independent processing makes inconsistent positions and number of non-zero coefficients in the target vector finally reconstructed in each channel, which is not favorable for extraction of target scattering information. For the improved OMP algorithm, the multi-channel target scattering information is used to determine candidate vectors and solve the projection coefficients of each channel in the same support set, so as to ensure the consistency of the position and number of non-zero coefficients in target vector reconstructed in each channel.

Setting of the preset threshold *Thres* is related to the noise level of the echo signal. Considering the high SNR ratio, the threshold can generally be set as about 0.05 of the energy of the echo signal. At this time, most of the scattering centers on the target can be accurately reconstructed; whereas, with the increase of noise in echo signal, the set threshold value also increases, so as to avoid more false scattering points caused by noise in the generated image. If the sparsity K is known, the reconstruction results obtained by applying the CS matching pursuit reconstruction algorithm are quite excellent but it is difficult to obtain accurate sparsity in actual engineering. In such case it always requires a large number of SAR images for statistical analysis to determine approximate sparsity K range of different kinds of observation scenes, and then obtain the optimal sparsity K within the determined range by the optimization criteria.

### 3.4. Extraction of Target Scattering Information

When the distance between the target and the antenna satisfies the near-field condition and the baseline of the antennas is much smaller than $R_{0}$, according to plane spectrum theory [29], the distance in Formula (1) can be represented as:

(25) $$R_{i}(t)=(R_{0}+r_{0}\cos(\theta_{0}+\omega t))\cos\alpha_{i}\cos\phi +r_{0}\sin(\theta_{0}+\omega t)\cos\alpha_{i}\sin\phi +(R_{0}\tan\alpha_{i}-z)\sin\alpha_{i},$$

where, $\phi =\arctan\left(\frac{r_{0}\sin(\theta_{0}+\omega t)}{R_{0}+r_{0}\cos(\theta_{0}+\omega t)}\right)$ is the angle between the target and antenna in the plane Oxy. To simplify the expression, the Cartesian coordinates of the target are represented in the cylindrical coordinate system, so Equation (25) can be expressed as:

(26) $$R_{i}(t)=\sqrt{{\left(R_{0}+r_{0}\cos(\theta_{0}+\omega t)\right)}^{2}+{\left(r_{0}\sin(\theta_{0}+\omega t)\right)}^{2}}\cos\alpha_{i}+(R_{0}\tan\alpha_{i}-z)\sin\alpha_{i}.$$

Then, the echo signal of the scattering point P can be represented as:

(27) $$s_{i}(t)=g_{i}\exp\left(-j4\pi f\frac{R\cos\alpha_{i}+(R_{0}\tan\alpha_{i}-z)\sin\alpha_{i}}{c}\right),$$

where, $R=\sqrt{{\left(R_{0}+r_{0}\cos(\theta_{0}+\omega t)\right)}^{2}+{\left(r_{0}\sin(\theta_{0}+\omega t)\right)}^{2}}$. This item is the same for the two antennas. According to Formula (26), it is found that phase information of the scattering point in target ISAR image contains height information of the scattering point, interferometric processing for two ISAR images is conducted, and the interferometric phase difference of P images is:

(28) $$\begin{array}{l} \Delta \varphi =\frac{4\pi f}{c}\left(R\cos\alpha_{2}+(R_{0}\tan\alpha_{2}-z)\sin\alpha_{2}-(R\cos\alpha_{1}+(R_{0}\tan\alpha_{1}-z)\sin\alpha_{1})\right)\\ \;=\frac{4\pi f}{c}\left(R(\cos\alpha_{2}-\cos\alpha_{1})+R_{0}(\tan\alpha_{2}\sin\alpha_{2}-\tan\alpha_{1}\sin\alpha_{1})-z(\sin\alpha_{2}-\sin\alpha_{1})\right) \end{array}$$

In the InISAR imaging system, the baseline length is much less than the distance between the antennas and target, so pitch angle difference between the antenna $TR_{1}$ and $TR_{2}$ is very small, namely $\alpha_{2}=\alpha_{1}+\Delta \alpha$, Δα≪1. In this paper, the antenna $TR_{2}$ is at the origin, so $\alpha_{1}=0$, $\alpha_{2}=\Delta \alpha$, and the height of the scattering point can be estimated as:

(29) $$z=\frac{\frac{\lambda }{4\pi }\Delta \varphi -R\cos\alpha_{2}-R_{0}\tan\alpha_{2}\sin\alpha_{2}}{-\sin\alpha_{2}}$$

When the antenna $TR_{1}$ and $TR_{2}$ are symmetrically distributed in the coordinate origin, namely $\alpha_{1}=\alpha_{2}$, the height of the scattering point is estimated as:

(30) $$z=\frac{\Delta \varphi \lambda }{8\pi \sin\alpha_{1}}$$

For target containing K scattering points, the height of each scattering point can be estimated by interferometric processing of the corresponding pixel points in the two ISAR images.

In actual processing, in order to avoid possible ambiguity of interferometric phase difference, the following judgements on the interferometric phase difference are required:

(31) $$\left\{ \begin{array}{l} if\Delta \varphi >\pi ,\Delta \varphi -2\pi \\ if\Delta \varphi <-\pi ,\Delta \varphi +2\pi \end{array}\right..$$

## 4. Experiments and Analysis

In order to verify the effectiveness of the algorithm in this paper, point target simulation data, electromagnetic software simulation data, and measured data in anechoic chamber is adopted to carry out imaging verification and performance analysis, respectively.

### 4.1. Numerical Simulations

**Figure 3:**
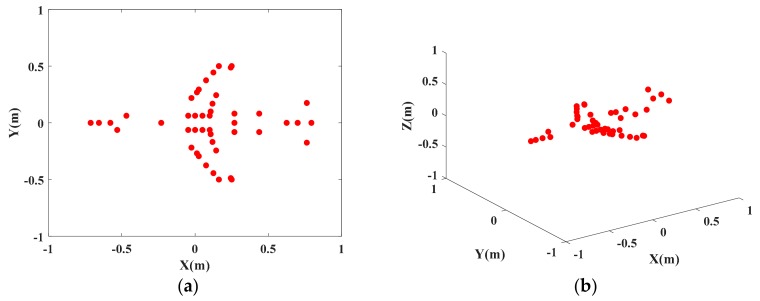
Geometric distribution for scattering model of plane point. (**a**) 2D distribution diagram of scattering point; (**b**) 3-D distribution diagram of scattering point.

The simulation target is composed of 46 scattering centers withe shape of plane model, and its distribution is shown in Figure 3. Stepped frequency signal of radar transmission and system parameters setting are as shown in Table 1.

**Figure 4:**
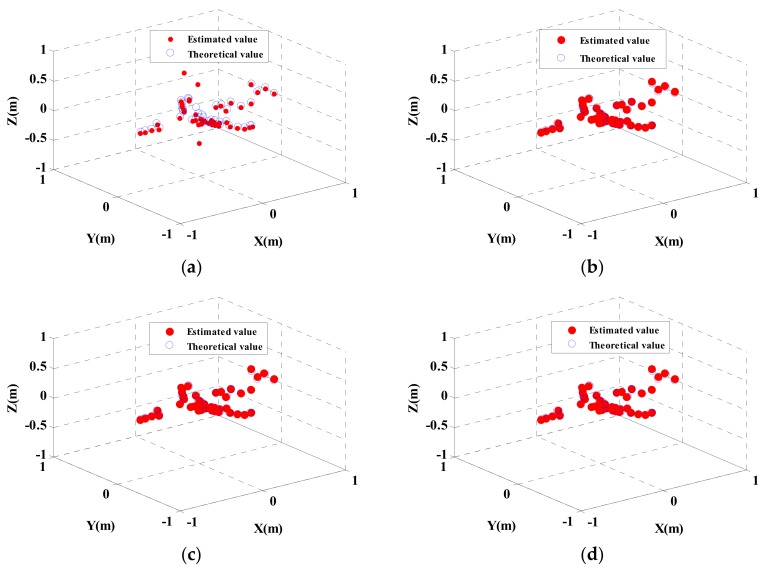
3-D Imaging results of near-field InISAR. (**a**) Traditional imaging approach, (**b**) Global sparsity mixed sum norm processing; (**c**) Global sparsity mixed Euclidean norm processing (**d**) Global sparsity mixed infinite norm processing.

Independent CS processing and joint CS processing based on global sparsity are separately used for InISAR imaging. The 3-D distribution of scattering points is shown in Figure 4. The above experiments are carried out with full data and signal without noise. Figure 4a is the single-channel independent processing reconstruction result, Figure 4b–d are the global sparse joint sparse reconstruction results. It can be seen from the figures that traditional single-channel independent processing can basically reflect the 3-D distribution of scattering points of the target, but cannot guarantee location consistency of all scattering points in different channels, which results in deviated location estimation. While with the proposed method, consistency of location and number of scattering points and more accurate reconstruction results can be ensured.

#### 4.1.1. Precision Analysis of Scattering Point Coordinate Estimation

Based on the above experiments, three typical scattering points are selected for statistical comparison. It can be seen from the coordinate values of scattering points in the Table 2 that the conventional imaging approach has obvious deviation in estimating the height coordinate values of scattering points, while the proposed approach is more accurate in estimating the location of scattering points. For example, when estimating the height information, the maximum deviation of the traditional imaging approach is 0.6438, while the maximum deviation of the approach in this paper is 0.046. Compared with the traditional method, the accuracy of the proposed approach is improved by roughly 90% as indicated by statistical analysis of the location errors of all scattering points.

#### 4.1.2. Precision Analysis of Interferometric Phase

**Figure 5:**
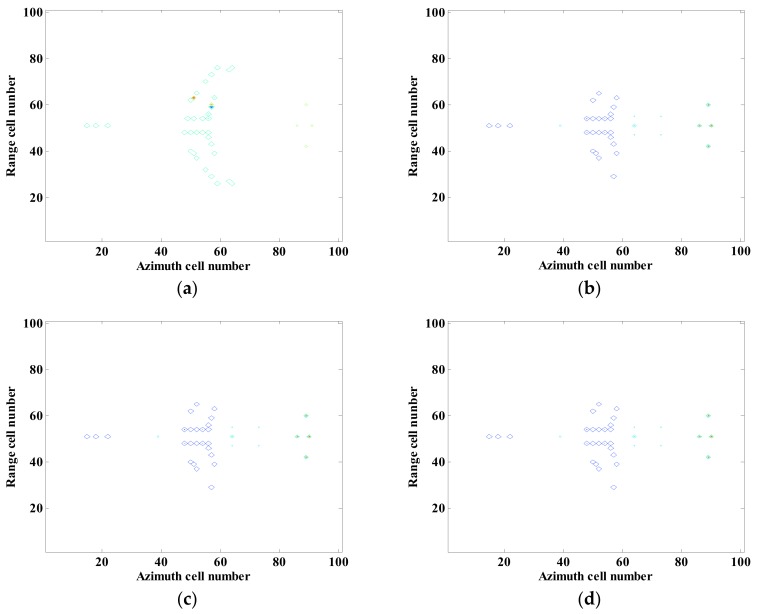
Interferometric phase image of near-field InISAR 3-D imaging. (**a**) Traditional imaging approach, (**b**) Global sparsity mixed sum norm processing; (**c**) Global sparsity mixed Euclidean norm processing (**d**) Global sparsity mixed infinite norm processing.

For near-field InISAR imaging system, to estimate the height information of target scattering point through the phase difference of corresponding pixel among complex images of various channels, it must ensure the consistency of strong scattering position among various channels images during imaging of various channels. The accuracy of interferometric phase has a direct influence on imaging quality. Figure 5 is the interferometric phase distribution of the complex image of two channels after processing. According to the comparative study on Figure 5a–d, the accuracy of scattering point in obtaining interferometric phase is higher and broader for the proposed approach comparing with that of the independent single-channel processing imaging approach.

#### 4.1.3. Noise Suppression Performance

**Figure 6:**
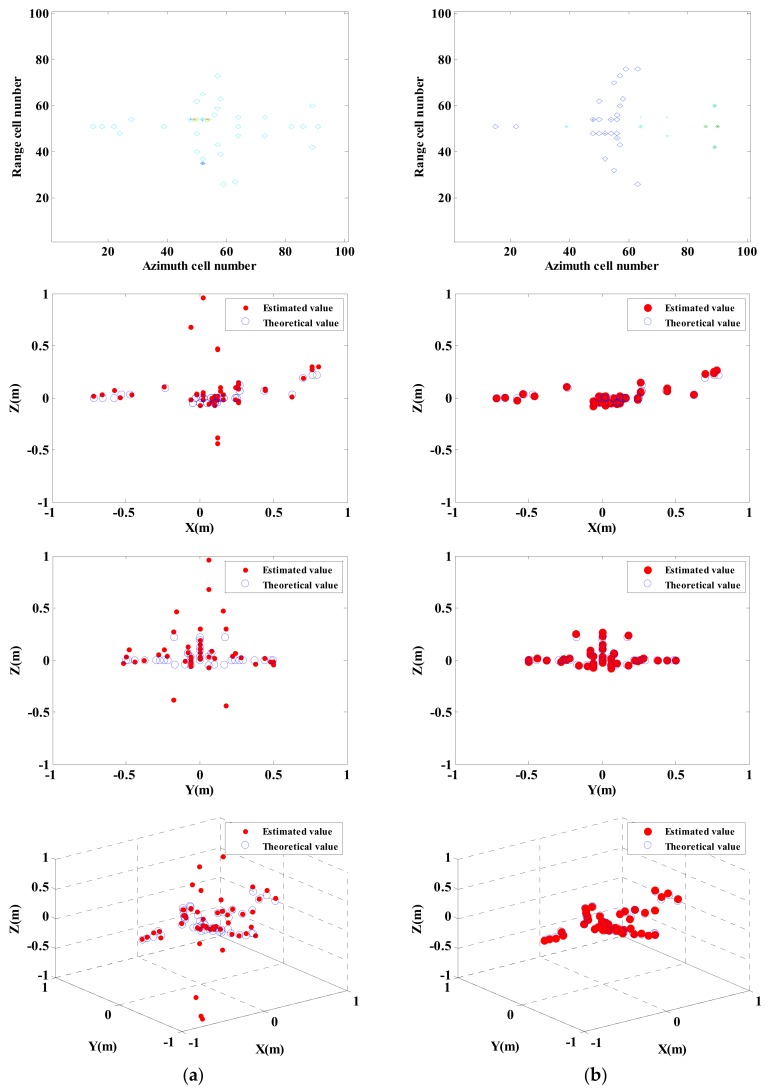
Imaging results obtained by adopting traditional approach and proposed approach respectively under 5 dB Noise. (**a**) Traditional imaging processing; (**b**) imaging processing of proposed approach (mixed Euclidean norm); interferometric phase images (top layer); complex images of channels 1 and 2 (second and third layers); final 3-D imaging results (last layer).

Figure 6 shows the results obtained by applying the traditional imaging approach and the proposed approach when there is noise in echo signal, where the noise is 5 dB complex white Gaussian noise. Add the complex white Gaussian noise with SNR ratio of −15 dB to 25 dB in the echo data, repeat Monte-Carlo simulation test for 100 times under every noise level, and calculate MSE estimated on the basis of height.

In Figure 6, imaging results show that there is a considerable deviation in estimation of traditional processing approach on height of target scattering point. While by applying the imaging approach (global sparsity Euclidean norm) proposed in this paper, the height of target scattering point can be estimated more accurate.

**Figure 7:**
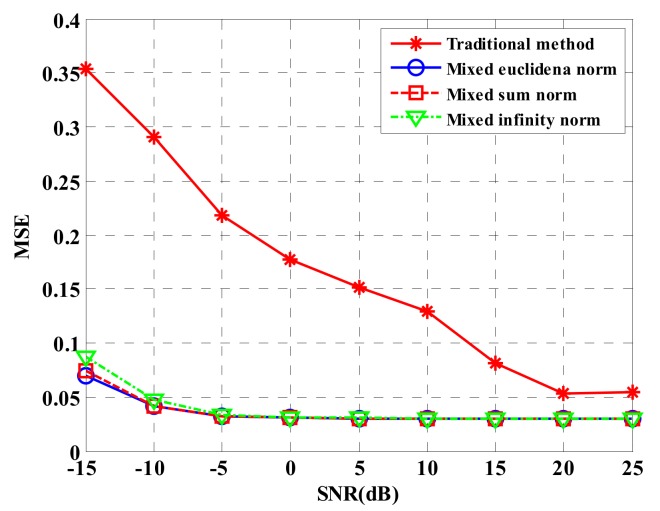
Comparison for imaging performance of four approaches under different SNRs.

Figure 7 describes MSE estimated by heights of four compressed sensing approaches under different SNRs. Under low SNR, the performance estimated by the heights of the four approaches is low. With the increase of SNR, height estimation performance increases. However, the estimation performance based on global sparsity in this paper is obviously superior to that obtained by the traditional processing approach.

#### 4.1.4. Performance of Sparsity Sampling Imaging

Randomly select a certain quantity of data from echo data to carry out InISAR imaging for further investigating the influence of sparsity sampling on height estimation. Then, estimate the height information of the scattering point through interference processing. The SNR is fixed as 10 dB, and the pitch angle of antenna TR1 is fixed as 0.05°. Carry out Monte-Carlo simulation for 100 times to every sparsity sampling scheme, and calculate the MES estimated on the basis of height.

**Figure 8:**
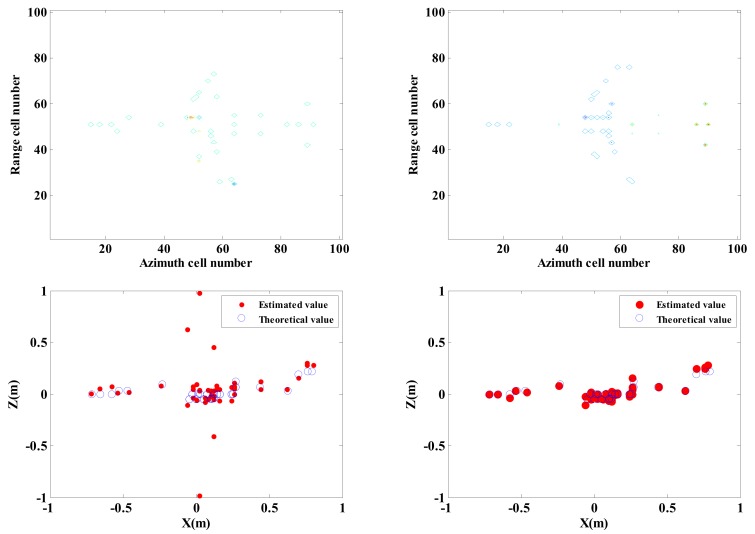

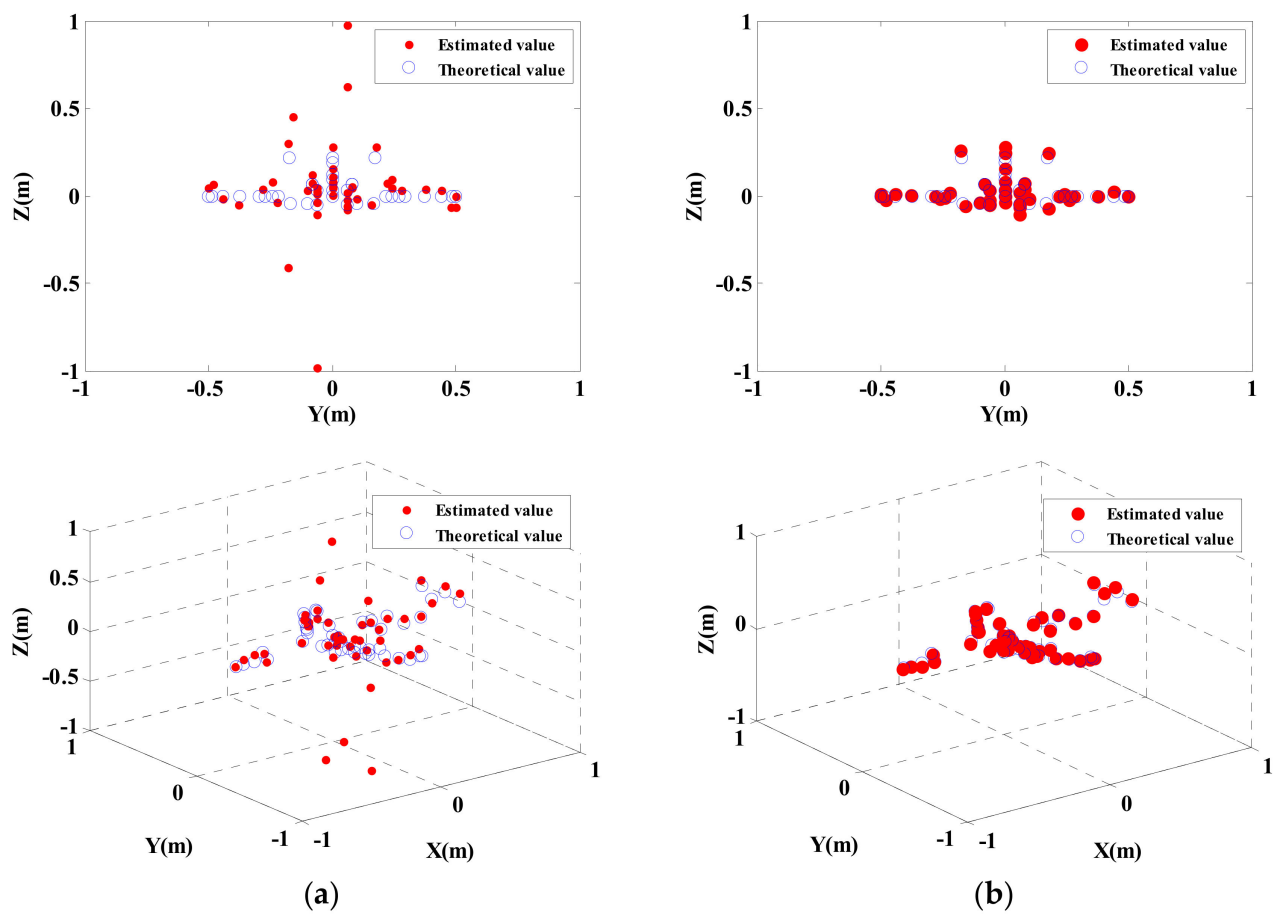
Imaging results of traditional approach and proposed approach under 20% of effective data. (**a**) Traditional imaging processing; (**b**) imaging processing of proposed approach (mixed Euclidean norm); interferometric phase images (top layer); complex images of channels 1 and 2 (second and third layers); final 3-D imaging results (last layer).

**Figure 9:**
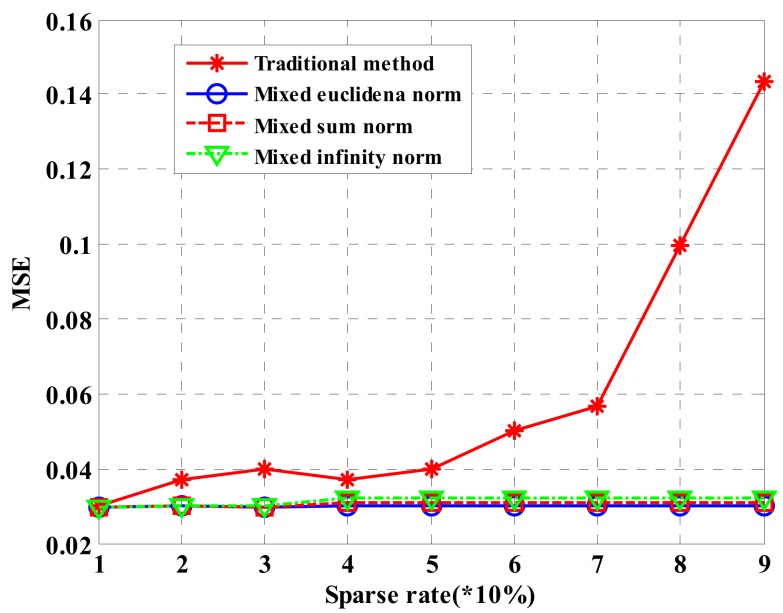
Comparison for imaging performance of four approaches under different sparsity samplings.

Figure 8 shows that application of the traditional approach fails to perform effective imaging of target given 80% under-sampling rate. However, with the proposed approach, accurate imaging of target can be performed. Figure 9 shows that the height estimation performance of processing based on global sparsity is still superior to that of results obtained on the basis of independent processing under sparsity sampling condition. Even when the measurement quantity is low (10% of full data), the overall sparsity constraint can still ensure a better interferometric imaging performance.

#### 4.1.5. Computational Complexity

The running time of independent CS approach of traditional single-channel depends on step (2). Its computing cost is $O\left(Lt_{end}N_{s}PQ\right)$, wherein, the $t_{end}$ is the times of algorithm iterative circulation, and the $N_{s}$ is the signal sampling times. The proposed approach has higher calculation efficiency and is only added with $O\left(Lt_{end}PQ\right)$ times of addition calculation compared to independent CS approach of single-channel. The increased calculation times by applying the proposed approach can be nearly ignored in practical application. On the basis of the space storage efficiency, the approach proposed in the paper needs to occupy more memory space compared to that needed by applying the independent CS approach of single-channel, but it can be effectively released through parallelization.

### 4.2. Experiments and Analysis of Backhoe

**Figure 10:**
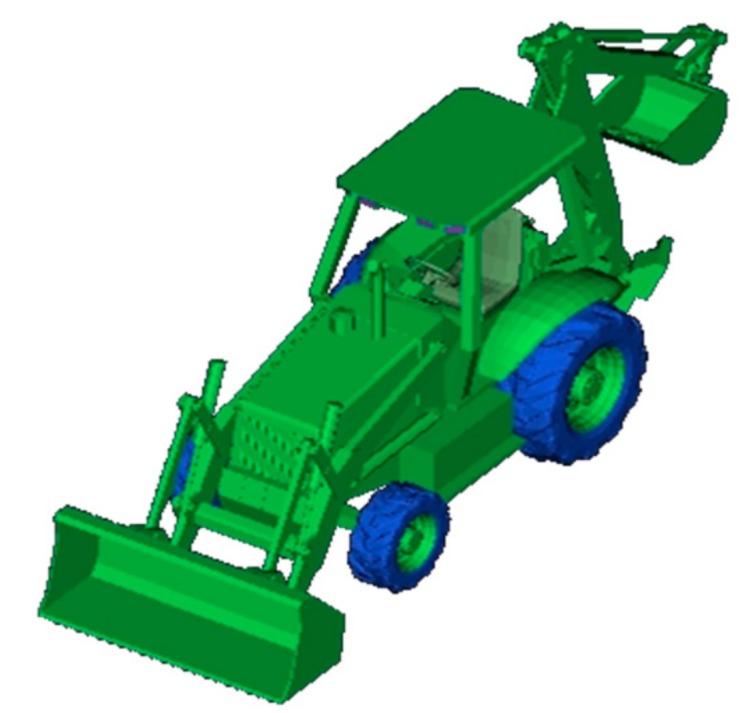
3-D CAD model of Backhoe.

The electromagnetic scattering echo data which is more closed to actual measurement is obtained by using high-frequency electromagnetic software and 3-D model of target. In this paper, Backhoe electromagnetic simulation data is adopted to verify the effectiveness of the proposed approach. In the experiment, select two groups of data with the adjacent pitch angle of 42° and 42.07° to divide the whole aperture into 17 sub-apertures. Specific parameters are shown in Table 3, and Figure 10 shows the CAD model of Backhoe.

**Figure 11:**
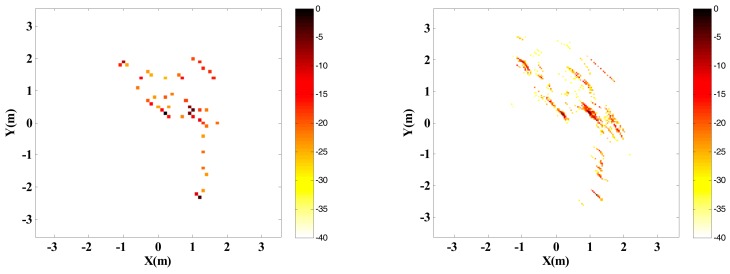

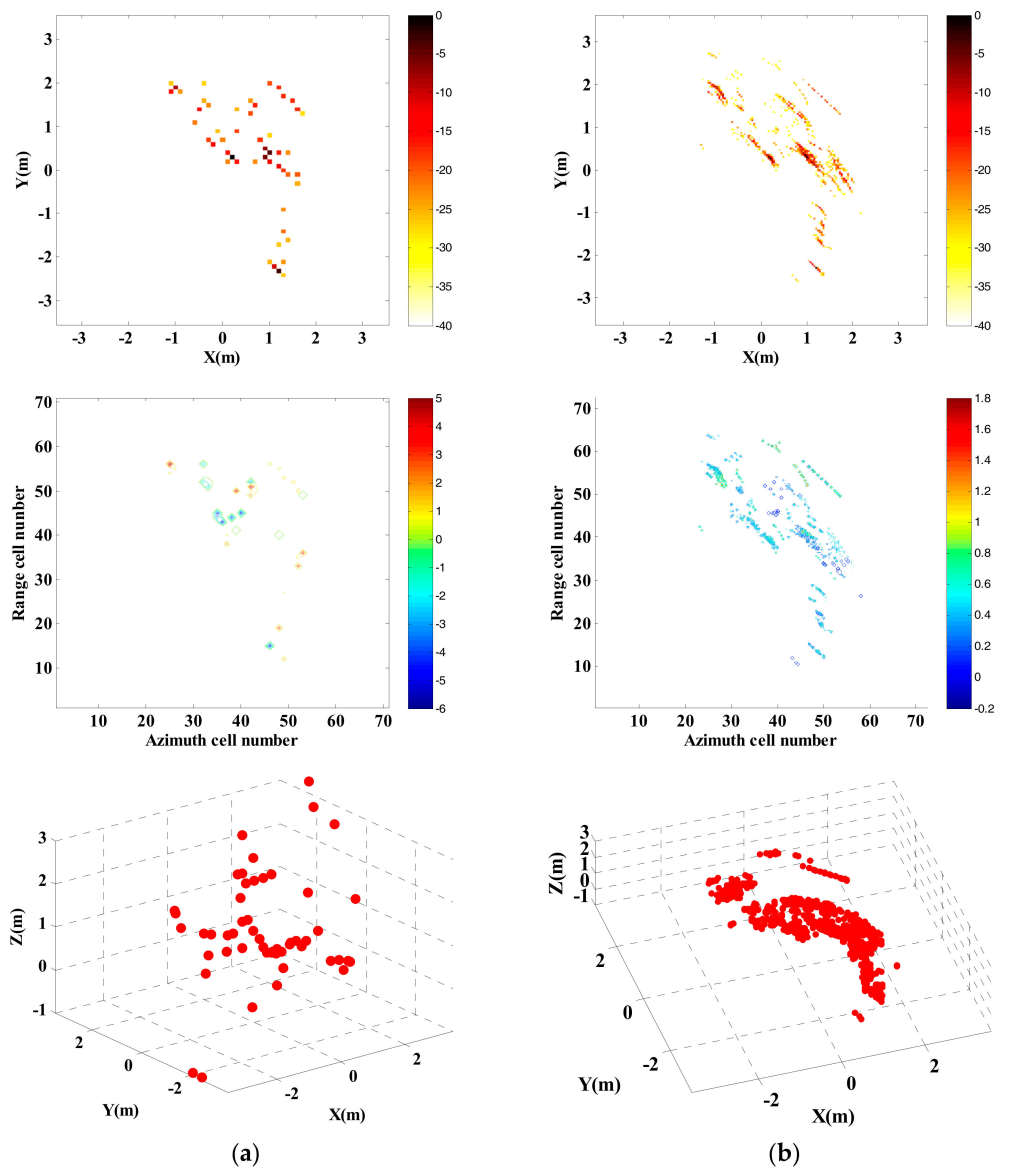
InISAR imaging results of Backhoe with complete data. (**a**) Traditional imaging processing; (**b**) imaging processing of proposed approach (mixed Euclidean norm); complex images of channels 1 and 2 (first and second layers); interferometric phase images (third layer); final 3-D imaging results (last layer).

Adopt traditional imaging approach and the proposed approach respectively to carry out InISAR imaging, and the 3-D distribution of target scattering points is shown in Figure 11.

**Figure 12:**
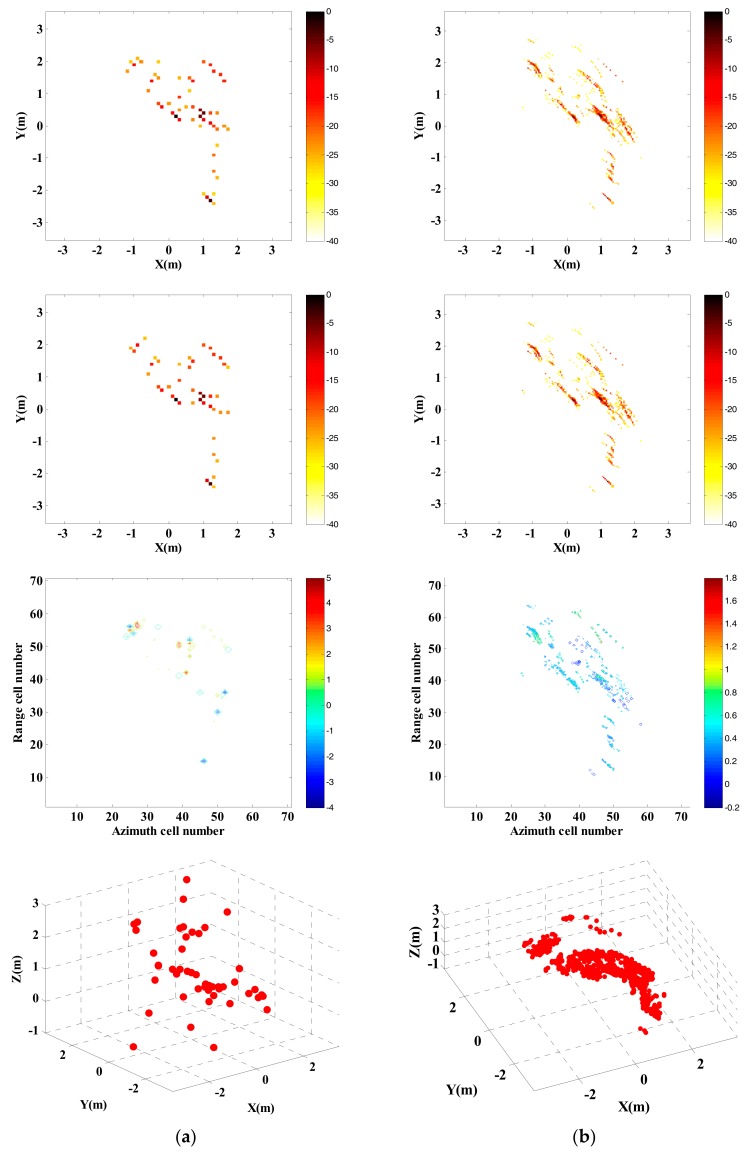
InISAR imaging results of Backhoe with 25% data. (**a**) Traditional imaging processing; (**b**) imaging processing of proposed approach (mixed Euclidean norm); complex images of channels 1 and 2 (first and second layers); interferometric phase images (third layer); final 3-D imaging results (last layer).

Randomly select 25% observed data from full data to generate the sparsity sampling data, and adopt traditional imaging approach and the proposed approach respectively to carry out InISAR imaging. The 3-D distribution of target scattering points is shown in Figure 12. Figure 11 and Figure 12 show that the reconstructed target information of traditional approach has a larger deviation. Regardless of complete data or insufficient data provided, the target can be imaged effectively. What’s more, with the decrease of data quantity, the imaging performance gets worse. On the contrary, by applying the proposed approach, 3-D target images with higher quality can be obtained. When the effective data is 25%, it can still maintain accuracy of height information in estimation.

### 4.3. Actual Measurement Experiment in Anechoic Chamber

**Figure 13:**
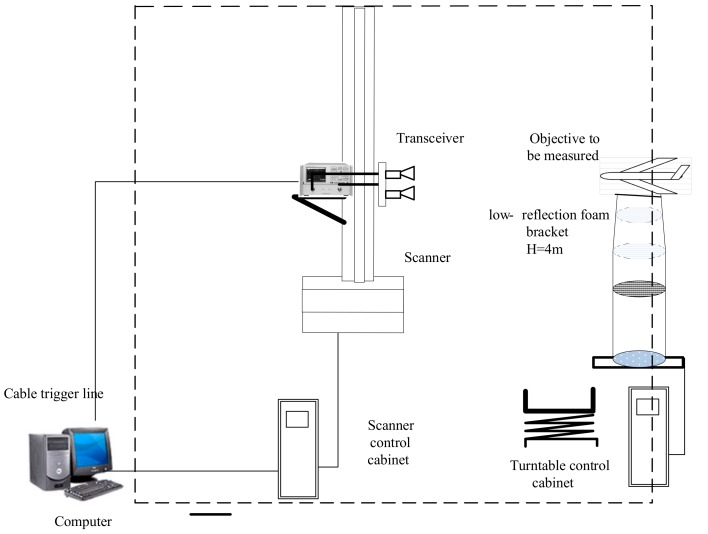
Frame diagram for near-field InISAR imaging system in anechoic chamber.

In order to further verify the effectiveness of the proposed approach in terms of practical applications perspective, a near-field InISAR test platform in an anechoic chamber is established, and the test system is shown as Figure 13. The target is put on the low scattering foam bracket, under which there is a turntable. The two antennas are fixed with interval of 0.2 m, and achieve azimuth accumulation through the rotation of turntable. Test parameters are shown in Table 4.

The parameter calculation rules are as follows:

(1) Antenna baseline

Height information of the target is mainly calculated by phase difference of dual-antenna propagation path. In general, the interferometric phase difference is a periodic function for the period with 2π. In order to avoid fuzzy height, the interferometric phase difference shall meet the requirement of Δφ≤2π, so that the baseline length meet the requirement of $d\leq \frac{\lambda R_{0}}{2H}$, wherein λ represents transmitting frequency, $R_{0}$ represents distance from the receiving/transmitting antenna to the target, and $\delta_{y}=\frac{c}{2B}$ represents maximum height of the target.

(2) Sampling principle

Range resolution in ISAR imaging is $\delta_{y}=\frac{c}{2B}$, and azimuth resolution is $\delta_{x}=\frac{\lambda }{2\theta }$, wherein B represents signal bandwidth and θ represents azimuth accumulation angle. In actual imaging, the range resolution is generally equal to the azimuth resolution. Concerning resolution requirements, the signal bandwidth and azimuth accumulation angle can be determined through $\delta_{y}=\frac{c}{2B}$ and $\delta_{x}=\frac{\lambda }{2\theta }$.

Concerning range resolution requirements, the frequency sampling interval is $\Delta f\leq \frac{c}{2R_{0}}$, also taken as step frequency interval.

Concerning azimuth resolution requirements, the azimuth sampling interval is $\Delta \theta \leq \frac{\lambda }{2D}$, wherein D represents maximum size of the target.

(3) Distance from antenna and target

This paper focuses on near-field imaging, in principle, distance from the antenna to the target is represented as: $R_{0}<\frac{4D^{2}}{\lambda }$, wherein D represents maximum size of the target and λ represents length of incident electromagnetic wave.

In addition, scanning in vertical direction does not exist because the two antennas are located fixedly, the beam center is fixed and only the target rotates in InISAR. As mentioned in Section 4.3, In the experiment, the range sampling interval and azimuth sampling interval are Δf′=nΔf(n=1,2,⋯,N) and Δθ′=mΔθ(m=1,2,⋯,M) respectively.

The test is adopted with stepped frequency signal, which features easy achievement of wideband and low requirements for hardware system. In order to make easier application of CS in the test, this paper adopts the deterministic sparsity observation approach based on Cat sequence for distance-oriented compressed sampling. The following shows the steps of Cat mapping to produce random sequence and construct observation matrix:

(1) Produce chaos sequence according to Cat mapping equation, and the mapping is defined as:

(32) $$\left[\begin{array}{c} x_{n+1}\\ y_{n+1} \end{array}\right]=\left[\begin{array}{cc} 1 & a\\ b & ab+1 \end{array}\right]\left[\begin{array}{c} x_{n}\\ y_{n} \end{array}\right]\left(\mathrm{mod}\;1\right),$$

where, (mod 1) represents the integer whose real number is casted out, namely x mod 1=x-⌊x⌋. $x_{n}$ sequence is selected to construct the needed deterministic random sequence.

(2) For using the chaos sequence construction $\Phi_{r}$ in stable area, cast out the g value in front of the sequence. It means to select $x_{g+1}$ as the starting point of the sampling. Meanwhile, sample the produced sequence with the interval of d for ensuring the mutual independence of elements in chaos sequence:

(33) $$z_{k}=x_{g+kd},k=0,1,2,\cdots ,N-1\;$$

After obtaining the output sequence $z_{k}$ of Formula (33), directly divide the $z_{k}$ into $N^{\prime }=N/U$ with equal interval. Select the corresponding position of maximum value in various intervals, and assign 1 to corresponding position of $\Phi_{r}$, and others a zero:

(34) $$\Phi_{r}=\left[\begin{array}{cccc} \underset{U}{\underset{︸}{1,\cdots ,0,\cdots ,0}} & 0 & \cdots & 0\\ \vdots & \ddots & \cdots & \cdots \\ 0 & 0 & \cdots & \underset{U}{\underset{︸}{0,\cdots ,0,\cdots ,1}} \end{array}\right],$$

where, each row has a (0,1) random sequence with length of U, and value 1 is at the position of the maximum value in the corresponding interval of the chaos sequence produced in {1,2,⋯,U}, and others are zero.

**Figure 14:**
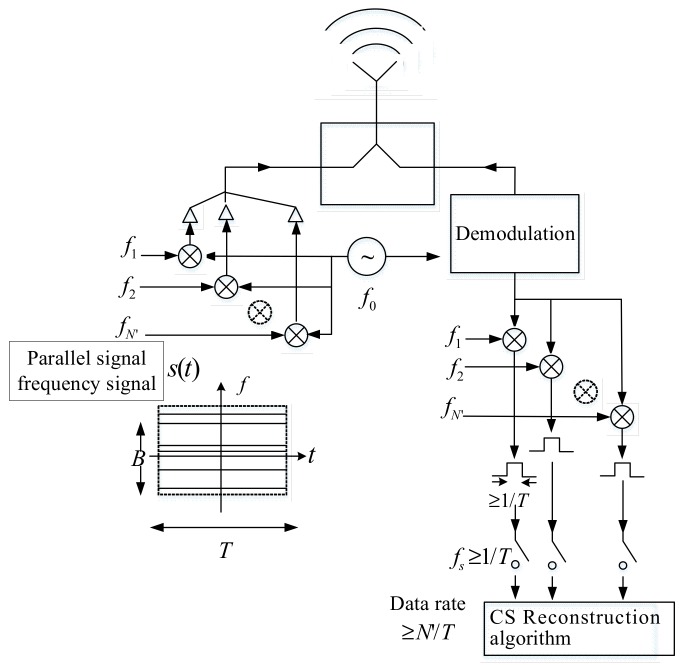
Range compressed sampling process of stepped frequency signal.

In practical applications, adopt parallelization to transmit $N^{\prime }$ random single frequency signal, and its data rate is $N^{\prime }/T$. Its achievement process is shown in Figure 14.

**Figure 15:**
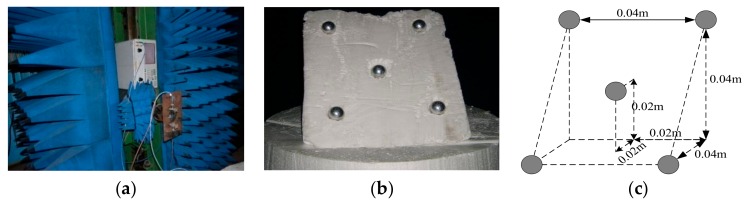
Target model of five metal balls. (**a**) Scanning frame and probe; (**b**) optical picture of five balls; (**c**) distribution of target spatial position.

**Figure 16:**
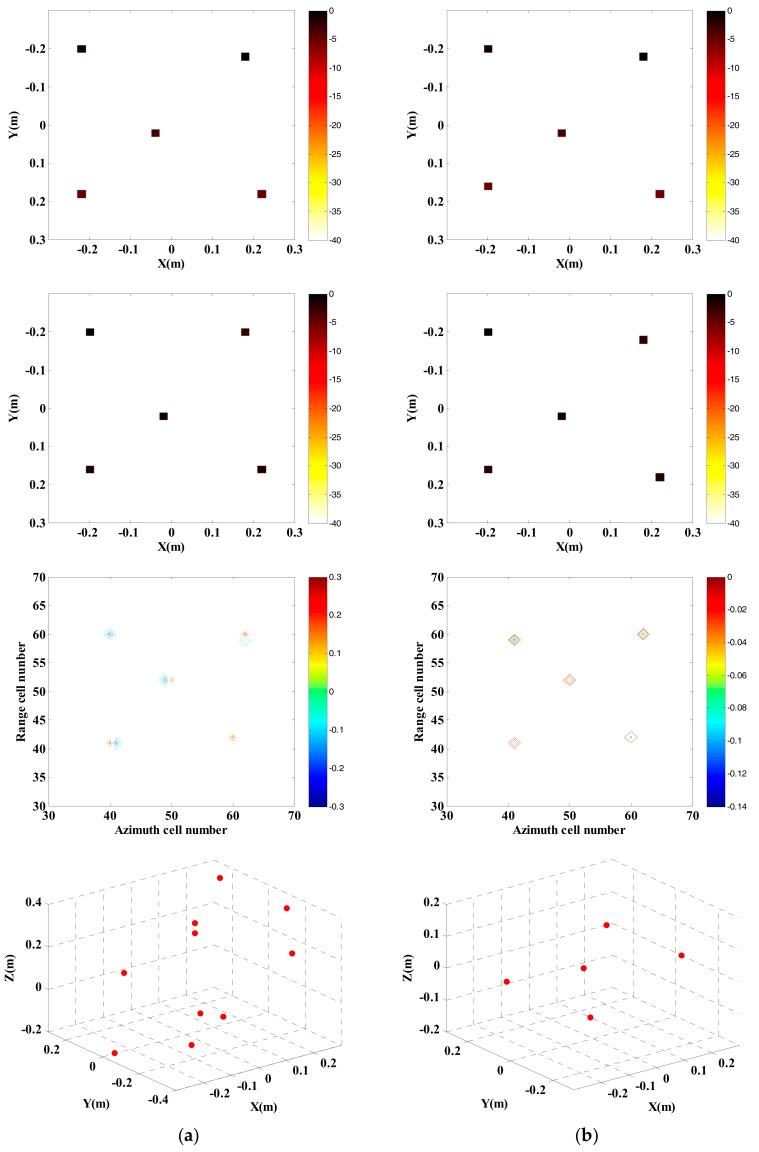
InISAR imaging results of five metal balls with complete data. (**a**) Traditional imaging processing; (**b**) imaging process of proposed approach (mixed Euclidean norm); complex images of channels 1 and 2 (first and second layers); interferometric phase images (third layer); final 3-D imaging results (last layer).

**Figure 17:**
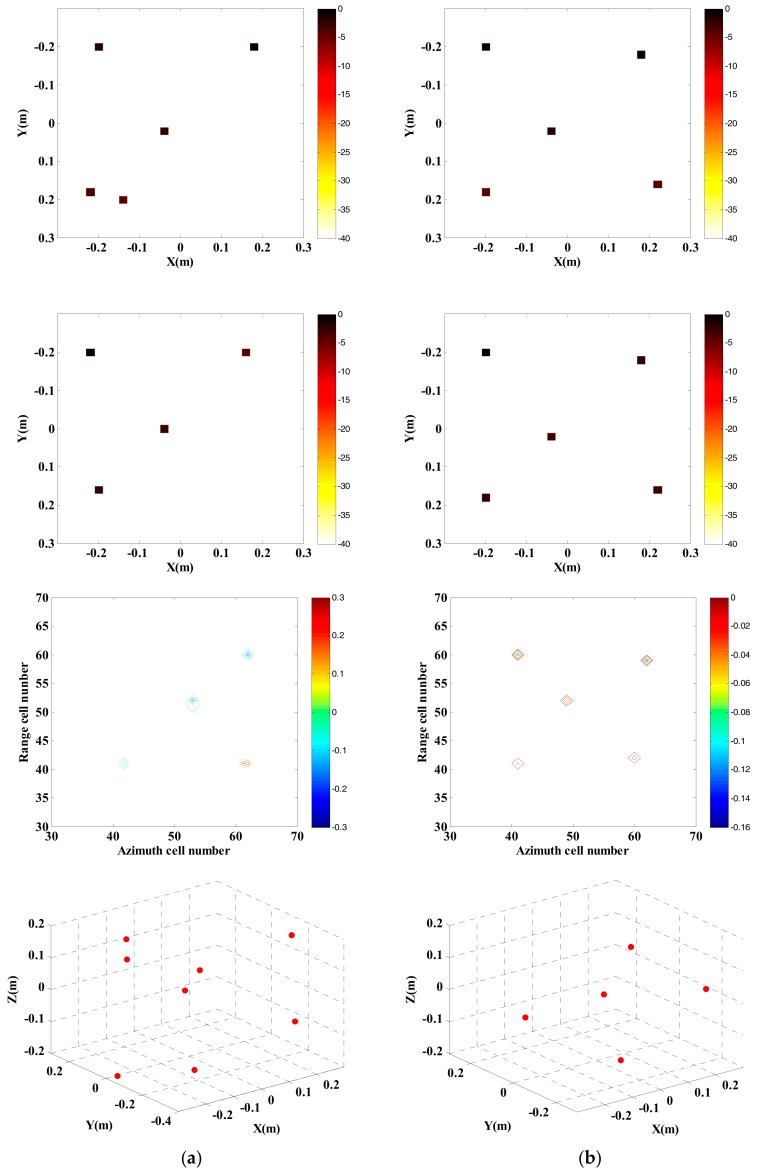
InISAR imaging results of five metal balls with 20% data. (**a**) Traditional imaging processing; (**b**) imaging processing of proposed approach (mixed Euclidean norm); complex images of channels 1 and 2 (first and second layers); interferometric phase images (third layer); final 3-D imaging results (last layer).

Figure 15 is the optical pictures of the measurement system and target distribution. Figure 16 and Figure 17 show imaging results by applying the two approaches provided with complete data and compressed sampling data. It can be found that by applying the traditional approach, target height information cannot be estimated completely because of incomplete correspondence of scattering point position, especially that the effective height information of target cannot be extracted if the compressed sampling proportion is large. Comparatively, by adopting the proposed approach, the height information of target can be estimated accurately given full data and compressed sampling data.

From Table 5 and Table 6, it can be found that by applying the traditional approach, position consistency of all scattering points in images of different channels cannot be ensured. If the compression rate is high, the consistency is more obvious. However, by applying the proposed approach, reconstruction is performed through multi-channel joint sparsity, so as to ensure that the scattering points are at the same pixel of the images from different channels, which is more favorable for extraction of target height information.

**Figure 18:**
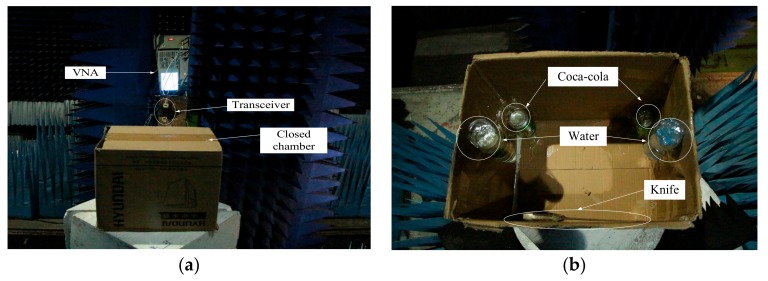
Imaging test for closed chamber. (**a**) Imaging system and target scene; (**b**) distribution of targets.

**Figure 19:**
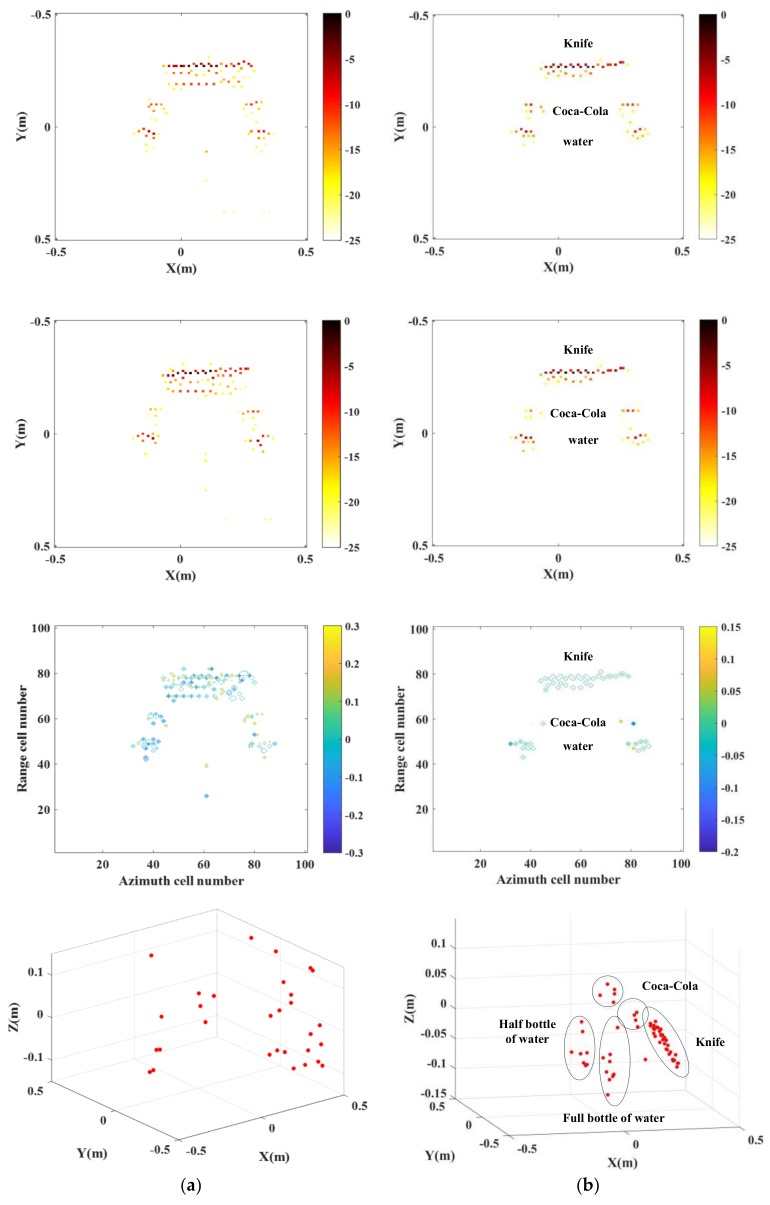
InISAR imaging results of anechoic chamber target with complete data. (**a**) Traditional imaging processing; (**b**) imaging processing of proposed approach (mixed Euclidean norm); complex images of channels 1 and 2 (first and second layers); interferometric phase images (third layer); final 3-D imaging results (last layer).

One of the practical applications of the proposed approach is security check. Here we conduct detecting and imaging on closed chamber in the anechoic chamber. The chamber is used to simulate luggage carrier, which is placed with one knife, two bottles of water (one is full and the other is half-full) and two bottles of Coco-cola. The test parameters are consistent with parameters as shown in Table 4. With 10% echo data adopted, Figure 18 shows the test system and target scene and distribution of targets within the box. The imaging results are as shown in Figure 19.

**Figure 20:**
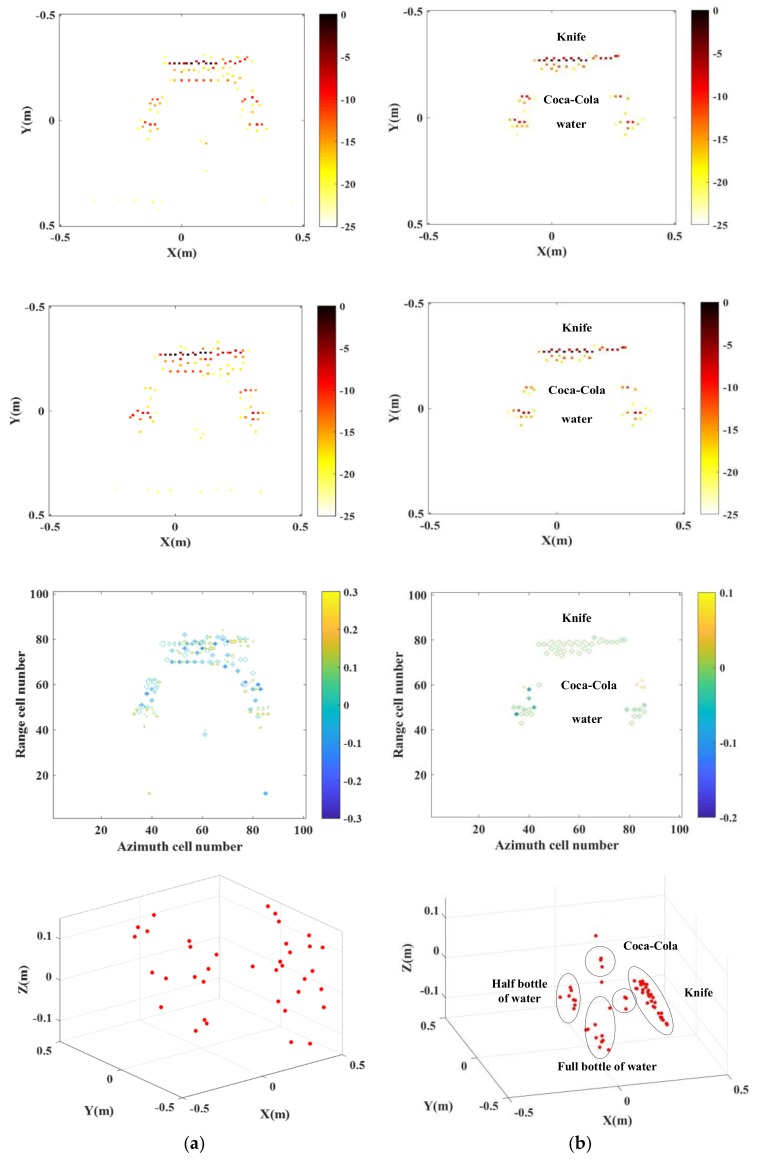
InISAR imaging results of anechoic chamber target with 20% data. (**a**) Traditional imaging processing; (**b**) imaging processing of proposed approach (mixed Euclidean norm); complex images of channels 1 and 2 (first and second layers); interferometric phase images (third layer); final 3-D imaging results (last layer).

It can be seen from Figure 20 that in the case of only 20% echo data applied, effective imaging on the targets is unable to be achieved with the traditional imaging approach. Through adoption of the proposed imaging approach, clear target images are available, provided with the shapes and location information of the knife, full bottle of water, half bottle of water and bottles of Coco-cola in the chamber.

## 5. Conclusions

Focusing on the near-field ISAR imaging, this paper puts forward an interferometric near-field 3-D imaging approach for joint sparsity reconstruction. Since scattering characteristics of targets in different channels are effectively made use of in joint sparsity, the imaging results feature a combination of interferometric processing and sparsity optimization. In addition to acquisition of near-field high-resolution 3-D images with less observation echoes applied, it can also accurately reflect the position information of scattering points. Moreover, it can effectively solve the problem that the accuracy of target scattering azimuth is not high in different directions by adopting sub-aperture synthesis. As verified by tests, target 3-D views with higher quality can be obtained by applying the imaging approach as proposed in this paper, so as to provide reliable judgment basis for target identification and other applications. Since this paper adopts an OMP-based reconstruction approach, the calculation complexity is not high. Also, it requires more research on rapid InISAR near-field 3-D imaging approach in combination with the traditional near-field imaging approach for future study.

## Figures and Tables

**Table 1:** Simulation parameter.

Parameter	Parameter Value
Carrier frequency	10 GHz
Bandwidth	4 GHz
Frequency step interval	40 MHz
Azimuth accumulation angle	20°
Azimuth sampling interval	0.2°
Distance between antenna and target	2 m
Baseline length	0.02 m

**Table 2:** Comparison of coordinates of typical scattering points.

	Scattering Point 1 (x, y, z)	Scattering Points 2 (x, y, z)	Scattering Points 3 (x, y, z)
Theoretical coordinate	(0.0125, 0.2688, 0)	(0.1438, 0.2438, 0)	(0.1188, 0.1688, 0.0467)
Traditional imaging	(0, 0.24, 0.6438)	(0.12, 0.23, 0.14)	(0.1, 0.1, 0.13)
Mixed sum norm	(0.009, 0.26, 0)	(0.1387, 0.24, 0.046)	(0.11, 0.1, 0.046)
Mixed infinite norm	(0.008, 0.26, 0)	(0.1387, 0.24, 0.046)	(0.1063, 0.1, 0.046)
Mixed Euclidean	(0.01, 0.265, 0)	(0.139, 0.241, 0.03)	(0.1163, 0.15, 0.046)

**Table 3:** Parameters of electromagnetic simulation system.

Parameter	Parameter Value
Carrier frequency	10 GHz
Bandwidth	6 GHz
Sampling point number of frequency	512
Azimuth accumulation angle	51°
Sampling point number of direction	71 * 17
Pitch angle	0.07°

**Table 4:** Parameters of near-field InISAR test system.

Parameter	Parameter Value
Carrier frequency	10 GHz
Bandwidth	4 GHz
Frequency step interval	40 MHz
Azimuth accumulation angle	20°
Azimuth sampling interval	0.2°
Distance between antenna and target	0.02 m
Baseline length	2 m

**Table 5:** Target position information provided with complete data.

Approach	Channel	Ball 1	Ball 2	Ball 3	Ball 4	Ball 5
Initial Traditional approach	--	(−0.20, −0.20)	(0.20, −0.20)	(0.00, 0.00)	(−0.20, 0.20)	(0.20, 0.20)
	1	(−0.22, −0.19)	(0.18, −0.18)	(−0.05, 0.02)	(−0.22, 0.19)	(0.22, 0.22)
	2	(−0.21, −0.18)	(0.18, −0.19)	(−0.03, 0.02)	(−0.22, 0.18)	(0.23, 0.23)
Proposed approach	1	(−0.20, −0.20)	(0.19, −0.19)	(−0.01, 0.01)	(−0.20, 0.19)	(0.21, 0.21)
	2	(−0.20, −0.20)	(0.19, −0.19)	(−0.01, 0.01)	(−0.20, 0.19)	(0.21, 0.21)

**Table 6:** Target position information provided with compressed sampling data.

Approach	Channel	Ball 1	Ball 2	Ball 3	Ball 4	Ball 5
Initial Traditional approach	--	(−0.20, −0.20)	(0.20, −0.20)	(0.00, 0.00)	(−0.20, 0.20)	(0.20, 0.20)
	1	(−0.20, −0.20)	(0.19, −0.19)	(−0.04, 0.01)	(−0.22, 0.19)	(−0.14, 0.20)
	2	(−0.21, −0.20)	(0.18, −0.19)	(−0.04, 0.00)	(−0.20, 0.17)	(Null, Null)
Proposed approach	1	(−0.20, −0.20)	(0.19, −0.19)	(−0.01, 0.01)	(−0.20, 0.19)	(0.20, 0.21)
	2	(−0.20, −0.20)	(0.19, −0.19)	(−0.01, 0.01)	(−0.20, 0.19)	(0.20, 0.21)
